# Incidence of sudden unexpected death in nocturnal frontal lobe epilepsy: a cohort study

**DOI:** 10.1016/j.sleep.2014.09.019

**Published:** 2015-02

**Authors:** Barbara Mostacci, Francesca Bisulli, Luca Vignatelli, Laura Licchetta, Lidia Di Vito, Claudia Rinaldi, Irene Trippi, Lorenzo Ferri, Giuseppe Plazzi, Federica Provini, Paolo Tinuper

**Affiliations:** a IRCCS Institute of Neurological Sciences of Bologna, Bologna, Italy; b Department of Biomedical and NeuroMotor Sciences, University of Bologna, Bologna, Italy; c Department of Primary Health Care, Bologna Health Trust, Bologna, Italy; d Regional Health and Social Agency, Emilia-Romagna Region, Bologna, Italy

**Keywords:** Nocturnal seizures, Obstructive sleep apnoea, Insular seizures, Generalised tonic-clonic seizures, Sleep

## Abstract

**Objective:**

Most cases of sudden unexpected death in epilepsy (SUDEP) follow a seizure, and most deaths occur while people are in bed, presumably sleeping. Nocturnal seizures are reported to be a risk factor for SUDEP. People with nocturnal frontal lobe epilepsy (NFLE) have seizures predominantly or exclusively during sleep, often many times per night. The present study aimed to assess whether NFLE represents a high-risk condition for SUDEP.

**Methods:**

The present study retrospectively assessed the incidence of SUDEP in a cohort reconstructed from a dedicated database of consecutive patients referred to the Epilepsy and Sleep Centres of the Institute of Neurological Sciences of Bologna from 1980 to 2012 with: (1) a diagnosis of NFLE, (2) at least 90% of seizures during sleep, and (3) at least one-year of follow-up.

**Results:**

One hundred and three people were included. The median time from seizure onset to last observation was 26 years, equal to a follow-up of 2789 person-years. One person died of SUDEP during the follow-up period. The incidence rate of SUDEP was 0.36 per 1000 person-years (95% CI 0.01 to 2.0).

**Conclusions:**

The incidence of SUDEP in the participant population was not higher than the rates previously reported in prevalent epilepsy populations (0.4 to 2.3 per 1000 person-years). The low prevalence of SUDEP might reflect the low occurrence of generalised tonic-clonic seizures in people with NFLE.

## Highlights

• We assessed sudden unexpected death in epilepsy in nocturnal frontal lobe epilepsy.
• The incidence of SUDEP in NFLE was no higher than that of other epilepsy populations.
• The lower than expected risk of SUDEP might reflect a low occurrence of GTCS in NFLE.

## 1 Introduction

The pathogenic mechanisms underlying sudden unexpected death in epilepsy (SUDEP) have not yet been unravelled. However, epidemiological studies have pointed to a number of risk factors and precipitating situational circumstances. A terminal seizure appears to be almost universal in the witnessed episodes and when the circumstances of death are reliably recreated [1–8]. Being found dead in bed is very common, especially in a prone position, presumably meaning that deaths occur preferentially during sleep, and the vast majority of the SUDEP cases recorded in video electroencephalography (EEG) monitoring units occurred at night [1,4–10]. Accordingly, a recent study suggested that a history of nocturnal seizures might be an independent risk factor for SUDEP [10].

Nocturnal frontal lobe epilepsy (NFLE) is a syndrome in which seizures occur mostly or exclusively during sleep and tend to be very frequent, occurring up to dozens of times per night [11]. Based on these assumptions, people with NFLE might be at higher risk of SUDEP. The aim of the present study was to assess the incidence of SUDEP retrospectively in a cohort of patients with NFLE, strictly defined as having more than 90% of seizures during sleep.

## 2 Methods

The present research is reported following the Strengthening the Reporting of Observational Studies in Epidemiology (STROBE) guidelines [12]. This retrospective cohort study was part of a larger study designed by the Istituto di Ricovero e Cura a Carattere Scientifico (IRCCS) of Neurological Sciences (INS) Bologna to investigate the features of NFLE. After approval by the local ethical (cod. 13084) committee, the study was conducted from November 2012 to December 2013.

### 2.1 Data sources

All patients attending the Epilepsy and Sleep Centres of the Institute of Neurological Sciences of Bologna between 1980 and September 2012 for paroxysmal sleep-related events compatible with frontal lobe seizures were progressively screened. The initial pool was partly reconstructed retrospectively by collecting historical, medical and video-polysomnographic (VPSG) records from the database of the Institute. A medical chart recording clinical and instrumental updates at every control visit or telephone/e-mail contact was available for each patient. Control visits in the centres were scheduled on an individual patient basis, depending on clinical needs, with intervals generally ranging from 6 to 12 months.

### 2.2 Cohort identification and data collection

Patients were eligible if they were diagnosed with NFLE, according to the following criteria: (1) a personal history of motor events arising predominantly from sleep, suggestive of a primary or secondary involvement of frontal lobe structures; (2) video-polysomnographic recording of one hyperkinetic/asymmetric tonic-dystonic episode or at least two stereotyped paroxysmal arousals (PA) [11,13].

Three experts in sleep medicine and epileptology (PT, FP, FB) confirmed the final diagnosis. All cases with VPSG recordings of stereotyped PA that were not associated with major ictal events were carefully reviewed and discussed collegially; the follow-up data were also considered, when available. The agreement required for the final diagnosis was 100%; otherwise these cases were considered doubtful and not included in the study.

Further inclusion criteria were: ≥90% of seizures occurring during sleep and within at least one year of follow-up. The lifetime rate of sleep seizures was independently assessed by BM and LD on the basis of the clinical records; doubtful cases were discussed and cases were only included when there was agreement. All data were retrospectively reviewed until last contact or death. Every effort was made to contact all of the patients who had their last visit prior to 2012 for a telephone interview aimed at verifying their current status and to update clinical information.

### 2.3 Terminology

Definite, probable and possible cases of SUDEP and SUDEP Plus were defined according to Nashef et al. [14].

### 2.4 Statistical analysis

The incidence of SUDEP was derived from the total number of people with SUDEP and the total person-year follow-up for the whole cohort. The 95% confidence interval (95% CI) was calculated according to the Poisson distribution.

## 3 Results

### 3.1 Population characteristics

**Fig. 1:**
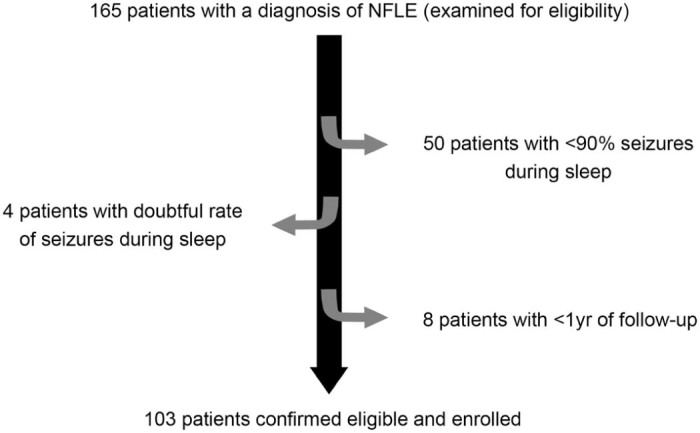
Flow diagram of the patients with NFLE.

In October 2013, the NFLE database contained 165 people. After reviewing the medical charts, 103 were judged to be eligible and included in the study (Fig. 1).

The median time from seizure onset to last observation was 26 years (range 2 to 81 years; mean 27 years; 25th percentile 18 years; 75th percentile 33 years), equal to a follow-up of 2789 person-years, counted from epilepsy onset to last observation/death. Among the people who did not have a check-up in the previous two years, 13 could not be reached by telephone due to a change in number, so their last contact preceded 2012. The median time since seizure onset to last observation in this subgroup was 23 years (range 10 to 39 years).

The study cohort comprised 70 males and 33 females. The mean age at onset of epilepsy was 15 years (range 0 to 53). The mean age at last observation was 43 years (range 9 to 86).

**Fig. 2:**
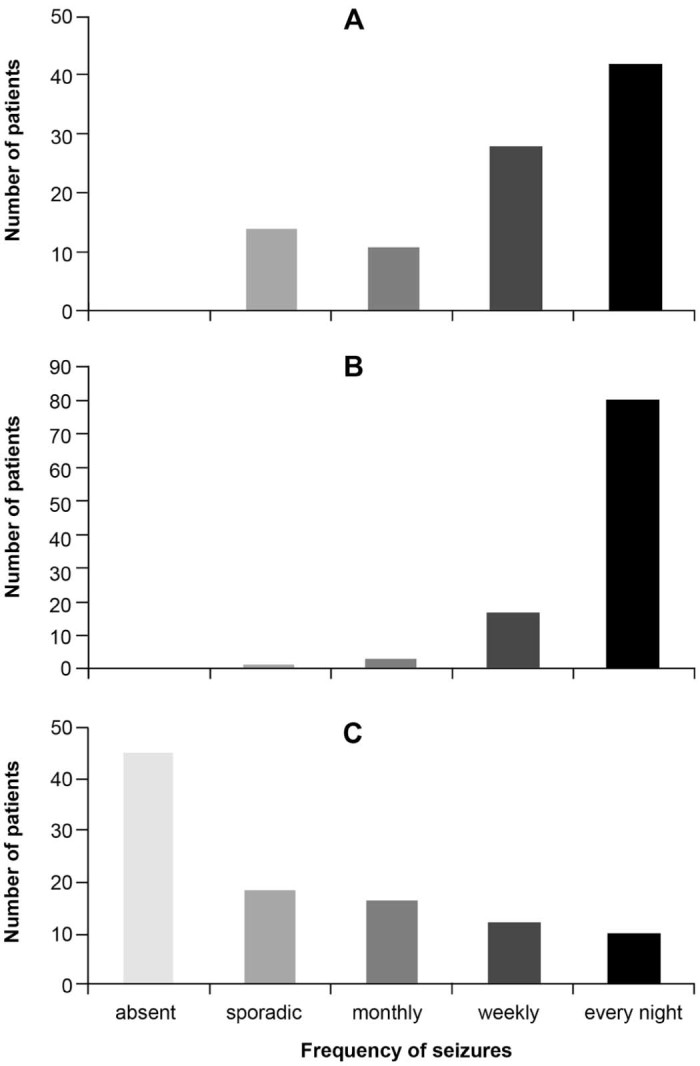
Frequency of seizures in the population. (A) Frequency at onset (data referred to 95 people, missing information in 8). (B) Peak frequency of seizures (101 people, 2 missing). (C) Frequency of seizures at the last observation (101 people, 2 missing).

The maximum lifetime frequency of seizures was every night for 80 people (77.7%), weekly for 17 (16.5%), monthly for three (2.9%), sporadic for one (1.0%) and unknown for two (1.9%). Frequency of seizures is represented in Fig. 2, as it was at onset (A), at their peak (B) and at last observation (C).

Twenty-nine people (28.1%) experienced secondary generalised tonic-clonic seizures (GTCS). This kind of seizure presented as a unique event or had a sporadic lifetime frequency in 17 people (16.5%), had a maximum lifetime frequency of one or more per year in six people (5.8%), a monthly maximum lifetime frequency in one person (1%), and an unknown maximum frequency in five people (4.8%).

Eight people (7.8%) had obstructive sleep apnoea (OSA), which was considered moderate-to-severe and required continued positive airway pressure (CPAP) treatment in four of them. In the remaining four, OSA was considered to be mild and a weight-loss programme was undertaken.

Eight people (7.8%) refused antiepileptic medication; 39 (37.8%) had been on monotherapy and 56 (54.4%) on polytherapy at some time during their lives. As a first line treatment, they were generally offered carbamazepine once a day, at night. Carbamazepine, alone or in combination, had been taken by 88 people (85.4%). Lamotrigine, alone or in combination, had been taken by 16 people (15.5%).

Ten people (9.7%) underwent a pre-surgical work-up: two subsequently underwent surgery (one removal of a left frontal Taylor dysplasia and the other a frontal corticectomy for an architectural dysplasia), three were judged to be inoperable after the pre-surgical work-up, three refused to undergo surgery or stereo-EEG, one improved significantly with the pharmacological therapy, and in one, the intervention was not performed due to significant surgical risk (see case description below).

A table listing the main characteristics of the people who were excluded because of <90% seizures during sleep is available as a supplementary file (Table 1).

### 3.2 Mortality rate and incidence of SUDEP

Two people died during the follow-up period. The mortality rate was 0.72 per 1000 person-years (95% CI 0.09 to 2.6). For one, the diagnosis was probable SUDEP (see SUDEP case description below). According to the newly proposed criteria [14], the diagnosis was reformulated into ‘Probable SUDEP Plus’. The other person (32 years old) died in a boat accident, in which there were witnesses: a terminal seizure and sudden death were reasonably excluded.

The incidence rate of SUDEP was 0.36 per 1000 person-years (95% CI 0.01 to 2.0).

### 3.3 SUDEP case description

The person was a 43-year-old man. His family history was unremarkable for epilepsy and sudden death; his father had chronic ischaemic heart disease. The man had severe OSA and was about to start CPAP therapy. He had an IQ of 71 and suffered from stuttering. His epilepsy started at the age of three. Seizures were characterised by right cephalic tingling or whistling in his right ear, subsequent tonic posturing of the right arm, right head turning, right eyelid myoclonus, sialorrhoea, and subsequent possible asymmetric tonic posturing of the inferior right limb and left arm. He could be conscious, but was unable to speak throughout the episode. The longest episodes tended to end with apnoea and myoclonus of all four limbs.

Since the age of 16, seizures had occurred almost exclusively during the night. His lifetime seizure frequency ranged from several seizures a week to dozens of seizures a night and he had been seizure-free at most for a month. He had experienced secondary generalised tonic-clonic seizures (GTCS), which reached their peak frequency between 21 and 23 years, when they were more than yearly. However, GTCS had not been reported for many years before his death. He proved to be drug-resistant for eight drugs, and at the time of his death he was on carbamazepine 1200 mg and topiramate 50 mg daily.

His EEGs showed very frequent interictal left fronto-centro-temporal spikes, enhanced during sleep, and ictal rhythmic activity in the same regions. An MRI examination disclosed a left insular-opercular Taylor dysplasia.

A stereo-EEG study, described elsewhere [15], identified the lesion as the site of ictal onset. Although excision of the lesion would have had a high chance of healing his epilepsy, the risk of subsequent language and motor deficits was considered to be unacceptable, as the lesion was in the insular region of the dominant hemisphere and very close to the pyramidal tract.

This man died during the night, while sleeping alone. He was found in prone. An autopsy was not performed.

## 4 Discussion

The incidence of SUDEP differs widely, depending mostly on the type of epilepsy population. The lowest rates are found in incident cohorts of epilepsy (0.09 to 0.35 per 1000 person-years) [16–18] and the highest are found in epilepsy surgery candidates or failures (6.3–9.5 per 1000 person-years) [18–20].

Sudden unexpected death in epilepsy occurs in bed at a higher frequency than that given by chance. A review by Nobili et al. reported that the mean percentage of possibly sleep-related SUDEP in studies including more than 10 subjects was 57%, reaching 95% in one [9].

The autonomic changes and fluctuations that are peculiar to sleep could interact with epilepsy, thus facilitating SUDEP [9]. Alternatively, sleep-related circumstances such as prone position and lack of surveillance could account for this relation. Recently, Lamberts et al. [10] conducted a case-control study comparing people with definite SUDEP to living controls with epilepsy. They found that SUDEP was prominently sleep-related (58% of the events) and that those who died were more likely to have a history of nocturnal seizures than the living controls. They concluded that nocturnal seizures might be an independent risk factor for SUDEP [10].

Surprisingly enough, the incidence rate of SUDEP in the present population with NFLE (0.36 per 1000 person-years) was lower than those previously reported in epilepsy clinic patients (1.08 to 5 per 1000 person-years) [21–24] and comparable to that of prevalent populations (0.4 to 2.3 per 1000 person-years) [18,25], even considering the highest limit of the confidence intervals (95% CI 0.01 to 2.0).

It is well established that GTCS are the most life-threatening kind of seizures. The occurrence of a GTCS shortly before death is a very frequent finding in witnessed and recorded SUDEP cases [1–7], as are clinical signs of terminal GTCS such as tongue biting [2]. A combined analysis of four case-control studies found that the presence and frequency of GTCS was the strongest single risk factor for SUDEP [26] and this was also confirmed after assessing the type and number of antiepileptic drugs and GTCS frequency concurrently [27]. GTCS preceded all 16 deaths and three of the five near-deaths in the MORTality in Epilepsy Monitoring Unit Study (MORTEMUS) series, which was a multicentric retrospective study analyzing all SUDEP and near-SUDEP recorded in 147 video-EEG monitoring units [8].

The present findings might depend on the low occurrence of GTCS in the studied population. Despite a high frequency of focal seizures, less than one third of the population experienced GTCS and most of them had only sporadic (or unique) episodes. This is in agreement with previous data showing that, although sleep has a more pronounced promoting effect on seizures arising from the frontal lobe than on seizures arising from other brain regions, it has a promoting effect on secondary generalisation of temporal and occipitoparietal, but not frontal seizures [28]. Even though it is established that seizures presenting with NFLE semiology could arise from other brain regions, the vast majority actually arise from the frontal lobe [29].

However, Lamberts et al. found that nocturnal seizures were still significantly associated with SUDEP, even after adjusting for the presence of GTCS in a multivariate analysis [10]. Therefore, other more elusive factors could account for this unexpected low incidence of SUDEP in NFLE. Because people with NFLE mainly have seizures while sleeping, supervision at night could be greater in this population than in epilepsies in which night seizures are occasional. Unfortunately, information on night supervision was not collected; however, this would have been difficult to procure over many years of follow-up at different ages. Prospective collection of this information could be useful.

The man who died from SUDEP had early onset refractory epilepsy with a high seizure frequency, all of which are ascertained risk factors for SUDEP. However, it is felt that the diagnosis should be reformulated as SUDEP Plus, according to the proposal by Nashef et al. [14], due to the OSA comorbidity. Obstructive sleep apnoea is thought to facilitate arrhythmias and has been proven to be an independent risk factor for sudden death, especially at night time [30,31]. This should be taken into account, as OSA was found in up to 7.8% of the present population, which is in agreement with previous reports of a high prevalence of OSA in people with epilepsy, particularly in refractory cases [32–34]. In addition, treating OSA could improve seizure control in comorbid people [35,36].

Furthermore, this man had seizures that originated in the insula, as demonstrated by a stereo-EEG study. Ryvlin described a similar patient, with insular epilepsy and nocturnal hypermotor seizures, who died from SUDEP [37]. Two further patients, recorded in video-EEG monitoring and described in the MORTEMUS study, had a near-SUDEP event following an insular seizure, one of them after an ictal asystole and the other after a postictal cardiorespiratory arrest [8]. Thus, due to the role of this region in cardiovascular control, seizures arising from the insula might carry a higher risk of SUDEP [37].

The present study was restricted to people with nocturnal seizures, which accounted for ≥90% of the total, in order to limit the observation to a subpopulation of NFLE who might be at highest risk of SUDEP, according to literature data. However, no SUDEP cases occurred in the 62 excluded cases.

## 5 Conclusions

In people with NFLE, there was no higher risk of SUDEP. According to the present study, which was conducted on a large cohort of patients with a rare syndrome characterised by seizures occurring predominantly or exclusively during sleep, and who were followed up for a long period, an additional factor may be required to make nocturnal seizures a risk factor for SUDEP. This could be the occurrence of GTCS.

## Conflict of interest

The ICMJE Uniform Disclosure Form for Potential Conflicts of Interest associated with this article can be viewed by clicking on the following link: http://dx.doi.org/10.1016/j.sleep.2014.09.019.

Conflict of interest ICMJE Form for Disclosure of Potential Conflicts of Interest form.

## Figures and Tables

**Table 1:** Demographic and clinical data of patients with <90% seizures during sleep.

N	50
Gender	M 58% F 42%
Median time from seizure onset to last observation	27 (Range 9–63)
Mean age at epilepsy onset	9 (Range 0–34)
Mean age at last observation	38 (Range 11–79)
Peak frequency of seizures	Every night 86.5% Weekly 10% Monthly 2.5% Unknown 1%
GTCS	48%
OSA comorbidity	4%
Therapy	None 8% Monotherapy 44% Polytherapy 48%
Surgery candidates	30% (Operated: 8%)
